# Regional Prevalence and Molecular Detection of *Enterocytozoon hepatopenaei* in Coastal Shellfish from Korea

**DOI:** 10.3390/ani15223356

**Published:** 2025-11-20

**Authors:** Beom Hee Lee, Eul Bit Noh, Hee Jung Choi, Mun Gyeong Kwon, Bo Seong Kim

**Affiliations:** 1 Department of Aquatic Life Medicine, Kunsan National University, Gunsan 54150, Republic of Korea; pm1646@naver.com (B.H.L.); nbiti7845@naver.com (E.B.N.); 2 Aquatic Disease Control Division, National Fishery Products Quality Management Service, Busan 46083, Republic of Korea; cchj77@korea.kr (H.J.C.); mgkwon@korea.kr (M.G.K.)

**Keywords:** *Litopenaeus vannamei*, *Venerupis philippinarum*, EHP carrier, *Crassostrea gigas*

## Abstract

EHP causes hepatopancreatic microsporidiosis in shrimp, leading to growth retardation without notable mortality. To examine potential environmental carriers, farmed bivalves were collected near shrimp ponds in Korea and screened for EHP using nested PCR targeting the spore-wall-protein gene. DNA of EHP was mainly detected in gill and digestive tissues of oysters, scallops, mussels, and clams. Histopathology after 24 h immersion exposure confirmed spores within the intestinal epithelium of clams, indicating short-term invasion potential. These results suggest that bivalves can retain and mechanically transfer EHP spores through shared seawater between shrimp farms and surrounding areas. Considering the environmental persistence of EHP, continuous surveillance of local invertebrates is recommended to reduce the risk of EHP introduction and further spread in aquaculture systems.

## Simple Summary

We detected *Enterocytozoon hepatopenaei* (EHP) from several farmed bivalves near shrimp ponds in Korea. EHP invaded the intestinal epithelium of clams within 24 h. Bivalves may serve as mechanical carriers with transmission potential for EHP between shrimp ponds and adjacent coastal waters. Regular monitoring of coastal shellfish is therefore recommended for early detection and preventing the spread of EHP in aquaculture.

## 1. Introduction

*Enterocytozoon hepatopenaei* (EHP) causes hepatopancreatic microsporidiosis, a chronic disease agent that suppresses shrimp growth and reduces yield [1,2]. Since its first detection, EHP has spread widely across Asia, including Korea [3,4,5]. The pathogen is transmitted horizontally through water and cohabitation [6] and may persist in various invertebrate carriers such as polychaetes, insects, false mussels, and crabs [7,8,9,10]. Economic losses in affected regions reach USD 232–567.62 million annually [1,2]. In Korea, EHP was first reported in Pacific whiteleg shrimp (*Litopenaeus vannamei*) in 2020 [3] and has since been detected in major shrimp-farming areas of Jeollabuk-do and Gyeongsangnam-do [5]. Because bivalves such as oysters, scallops, mussels, and clams are cultivated near shrimp ponds, shared seawater systems may expose them to EHP spores. Although some bivalves have been experimentally identified as potential carriers [10], field data from Korea remain limited. This study therefore investigated the prevalence of EHP in farmed bivalves and further examined whether Manila clams, as a representative species, could act as short-term mechanical carriers capable of transmitting EHP to shrimp under experimental conditions. The findings provide baseline information to support biosecurity and disease-management strategies for shrimp aquaculture.

## 2. Materials and Methods

### 2.1. Sampling

Sampling was conducted in major aquaculture regions of Korea to detect EHP in farmed bivalves, reflecting the regional production distribution of each species. For each region, one representative farm was randomly selected, and ten market-sized individuals were collected from harvested stocks to minimize sampling bias. Bay scallops, Pacific oysters, Manila clams, and Mediterranean mussels were obtained from their principal farming areas and processed for molecular detection (Table 1). Although the sample size was limited, this design allowed a preliminary assessment of EHP prevalence in representative species.

### 2.2. PCR and qPCR Assays for EHP Detection and Prevalence

Tissues including gill, digestive tubule, mantle, stomach, and intestine tissues were dissected from each specimen. Genomic DNA was then extracted from each organ using the QIAamp^®^ DNA Mini Kit (Qiagen, Hilden, Germany) following the manufacturer’s instructions.

To detect EHP, nested PCR (SWP-PCR) was performed as previously reported [11], using highly sensitive and specific primer sets [12]. In addition, TaqMan-based quantitative PCR (qPCR) targeting the small subunit ribosomal DNA (SSU rDNA) was conducted using primer set F157/R157 with a detection limit as low as 4 × 10^1^ copies per reaction [13].

For qPCR standard curve preparation, a 157 bp PCR amplicon generated from the hepatopancreas of EHP-infected shrimp using the published primer set [13] was cloned into a pMG-Amp vector by Macrogen Inc. (Seoul, Republic of Korea). The recombinant plasmid was 2867 bp in size and had a concentration of 10.2208 ng/μL, corresponding to 3.30 × 10^9^ copies/μL. A standard curve was generated using a ten-fold dilution series (3.30 × 10^3^ to 3.30 × 10^5^ copies/μL), yielding an R^2^ value of 0.998. Each qPCR sample was analyzed in duplicate to ensure reproducibility. Differences in qPCR values across regions were analyzed using Kruskal–Wallis test, and post hoc comparisons were conducted using Conover’s test (*p* < 0.05).

EHP prevalence at shellfish farms was estimated from nested PCR results using the “prevalence (version 0.4.1)” package in R (version 4.5.1), with test sensitivity and specificity set at 95–100% [3].

### 2.3. Histopathological Analysis of EHP-Containing Manila Clam

To confirm the invasion and presence of EHP in Manila clams, as a representative shellfish, seven clams were immersed in 10 L of seawater (30 ppt, 23 °C) containing EHP spores at a concentration of 2.8 × 10^6^ cells/L. After 2 h, Shellfish Diet 1800 (Instant Algae, Reed Mariculture, Campbell, CA, USA) was provided according to the manufacturer’s instructions. The clams were exposed for 24 h, rinsed three times with seawater, and fixed in 10% neutral buffered formalin for 24 h. Subsequently, standard histopathological procedures were performed to obtain 5 µm-thick tissue sections, which were stained with Giemsa and examined microscopically.

### 2.4. Transmission of EHP to L. vannamei After Feeding on EHP-Exposed Manila Clams

EHP-exposed Manila clams were transferred to fresh seawater (10 L; 30 ppt; 23 °C) in a new tank at 24 h intervals for three consecutive days, with Shellfish Diet 1800 provided once daily. On day 3, clams were rinsed, and the visceral mass was extracted and fed to three EHP-free *L. vannamei* maintained in 20 L of seawater. For the control group, clams were exposed to phosphate-buffered saline (PBS) instead of EHP under identical conditions before feeding to shrimp. Five days post-feeding, the hepatopancreas of each shrimp from both groups was sampled and subjected to SWP-PCR for EHP detection.

## 3. Results

### 3.1. Regional Sampling Results of SWP-PCR and qPCR

EHP was detected in all four shellfish species across the surveyed regions, showing clear regional and species-specific variation (Table 2). Based on SWP-PCR results, high detection rates (>70%) in digestive or gill tissues were observed in Bay scallops, Manilla clams, and Pacific oysters. qPCR analysis revealed significant regional variation in EHP copy numbers. The Kruskal–Wallis confirmed these differences (*p* = 0.0007), and Conover’s post hoc analysis indicated that Goseong-gun showed significantly higher EHP copy numbers compared to Boryeong-si, Yeosu-si, and Tongyeong-si (Table 2).

### 3.2. Histopathological Analysis of EHP-Exposed Manila Clams

**Figure 1:**
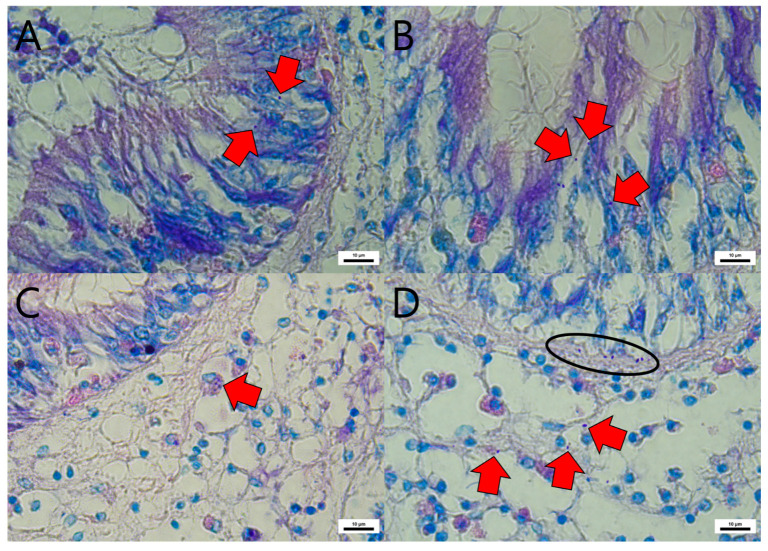
Histopathological observations of Manila clams after experimental EHP exposure via 1-day immersion, stained with Giemsa and EHP (red arrows) observed in the cytoplasm of the midgut epithelium (**A**,**B**), Hemocyte engulfing EHP (red arrow) in the connective tissue beneath midgut epithelium (**C**), EHP beneath the epithelial cells (ellipse) of midgut and disseminated in connective tissue (**D**).

In EHP-exposed Manila clams, spores measuring approximately 1.4 μm in length were observed in the intestinal region. EHP was localized within the cytoplasm of midgut epithelial cells (Figure 1A,B) as well as in the basement membrane and connective tissue underlying the midgut epithelium (Figure 1D). Additionally, some hemocytes situated beneath the intestinal epithelium were found to have engulfed EHP spores (Figure 1C).

### 3.3. Transmission of EHP L. vannamei After Feeding on EHP-Containing Manila Clams

In the group of *L. vannamei* fed EHP-exposed Manila clams, SWP-PCR detected EHP in 33% of individuals (1/3). In contrast, all shrimp fed Manila clams exposed to phosphate-buffered saline (PBS) tested negative (0/3).

## 4. Discussion

The results of this study provide new evidence that EHP persists in Korean aquaculture environments and can interact with non-shrimp species. Detection of DNA of EHP in multiple bivalve species indicates that the pathogen circulates beyond shrimp populations and may be sustained by surrounding filter-feeding organisms.

Because bivalves filter particles within 1–30 µm, overlapping the spore size of EHP (1.4–1.7 µm) [1,11], they can readily trap and retain spores released from shrimp ponds. Accumulated spores may subsequently be discharged through excretion, decay, or effluent water during shellfish processing, promoting local contamination. In coastal Korea, where shrimp and shellfish farms often share seawater systems, such exchange could provide a continuous route of exposure and reinfection.

This study demonstrated EHP presence in all four examined bivalve species and histological localization within the intestinal epithelium and connective tissue of Manila clams after 24 h of exposure. Feeding experiments confirmed that these clams could transmit EHP to *L. vannamei*, providing direct evidence that bivalves act as short-term mechanical carriers. Although based on limited sample size and duration, the consistent detection of EHP in bivalves collected near major shrimp-farming areas supports the hypothesis that coastal shellfish contribute to pathogen maintenance and dissemination.

Given the high environmental resilience of EHP spores due to their chitinous wall [14,15,16,17], management strategies should emphasize continuous surveillance of local invertebrates, effluent control, and separation of seawater systems between shrimp and shellfish farms. Such integrated biosecurity measures are essential for early detection and prevention of EHP introduction into shrimp aquaculture.

## 5. Conclusions

In conclusion, this study confirmed that bivalves located near Pacific whiteleg shrimp farms frequently harbor EHP, with Manila clams showing particularly high detection rates across multiple organs. Short-term EHP exposure experiments demonstrated the localization of EHP within the intestinal epithelium and connective tissue, suggesting a potential route for systemic dissemination. The environmental persistence of EHP spores indicates that bivalves may act as mechanical carriers, posing a transmission risk in aquaculture environments. Regular monitoring of surrounding organisms is essential for early detection and prevention of EHP introduction into shrimp farms.

## Figures and Tables

**Table 1:** Summary of sampling for EHP detection in farmed shellfish (Pacific Oysters, Bay Scallops, Manila Clams, and Mediterranean Mussels) in various administrative regions in Korea. Shell length and weight are presented as mean ± standard deviation (SD).

Province	Site	Organism	Sampling Date	Shell Length (cm)	Weight (g)	Number of Sampled Individuals
Gyeonsangnam-do	Goseong-gun	Bay scallop	March 2024.	6.7 ± 0.6	43.9 ± 9.1	10
		Pacific oyster	March 2024.	12.4 ± 0.9	125.9 ± 21.5	10
	Tongyeong-si	Bay scallop	February 2024.	6.7 ± 0.7	37.4 ± 11.6	10
		Pacific oyster	February 2024.	10.0 ± 1.5	63.6 ± 24.6	10
Jeollanam-do	Goheung-gun	Manila clam	February 2024.	4.7 ± 0.3	11.5 ± 2.6	10
	Yeosu-si	Mediterranean mussel	March 2024.	6.6 ± 0.6	14.6 ± 4.4	10
Chungcheongnam-do	Boryeong-si	Pacific oyster	March 2024.	9.5 ± 0.9	77.1 ± 19.8	10

**Table 2:** Detection of EHP in farmed shellfish by region and species in Korea using SWP-nested PCR and qPCR. Superscripts next to the region names indicate statistically significant differences in qPCR values among regions, as determined by Kruskal–Wallis test followed by Conover’s post hoc analysis (*p* < 0.05).

Province	Site	Species	Method	Organs					Average Prevalence (95% CI)
				Mantle	Digestive Gland	Gill	Stomach	Intestine	
Gyeonsangnam-do	Goseong-gun ^a^	Bay scallop	SWP-PCR	40% (4/10)	10% (1/10)	70% (7/10)	20% (2/10)	70% (7/10)	67.4% (37.9~91.3%)
			qPCR	- (0/10)	- (0/10)	3.0 × 10^2^ copies (2/10)	- (0/10)	2.2 × 10^2^ copies (2/10)	
		Pacific oyster	SWP-PCR	30% (3/10)	30% (3/10)	20% (2/10)	20% (2/10)	30% (3/10)	32.2% (8.7~60.9%)
			qPCR	2.7 × 10^2^ copies (1/10)	7.8 × 10^2^ copies (1/10)	2.8 × 10^2^ copies (1/10)	1.5 × 10^2^ copies (1/10)	2.2 × 10^2^ copies (2/10)	
	Tongyeong-si ^b^	Bay scallop	SWP-PCR	0% (0/10)	80% (8/10)	80% (8/10)	50% (5/10)	50% (5/10)	76.2% (47.8~96.2%)
			qPCR	- (0/10)	- (0/10)	- (0/10)	- (0/10)	- (0/10)	
		Pacific oyster	SWP-PCR	10% (1/10)	50% (5/10)	30% (3/10)	60% (6/10)	20% (2/10)	58.8% (29.5~85.3%)
			qPCR	- (0/10)	- (0/10)	- (0/10)	- (0/10)	- (0/10)	
Jeollanam-do	Goheung-gun ^a,b^	Manilla clam	SWP-PCR	70% (7/10)	60% (6/10)	60% (6/10)	70% (7/10)	80% (8/10)	76.2% (47.8~96.2%)
			qPCR	4.0 × 10^2^ copies (1/10)	2.9 × 10^2^ copies (1/10)	- (0/10)	- (0/10)	2.2 × 10^2^ copies (1/10)	
	Yeosu-si ^b^	Mediterranean mussel	SWP-PCR	10% (1/10)	40% (4/10)	40% (4/10)	40% (4/10)	10% (1/10)	41.4% (15.1~71.1%)
			qPCR	- (0/10)	- (0/10)	- (0/10)	- (0/10)	- (0/10)	
Chungcheongnam-do	Boryeong-si ^b^	Pacific oyster	SWP-PCR	30% (3/10)	50% (5/10)	80% (8/10)	30% (3/10)	50% (5/10)	76.2% (47.8~96.2%)
			qPCR	- (0/10)	- (0/10)	- (0/10)	- (0/10)	- (0/10)	

## Data Availability

All data are contained within the article. Please contact the corresponding author for additional data requests.

## References

[1] Shinn A.P., Pratoomyot J., Griffiths D., Trong T.Q., Vu N.T., Jiravanichpaisal P., Briggs M. (2018). Asian shrimp production and the economic costs of disease. Asian Fish. Sci..

[2] Patil P.K., Geetha R., Ravisankar T., Avunje S., Solanki H.G., Abraham T.J., Vinoth S.P., Jithendran K.P., Alavandi S.V., Vijayan K.K. (2021). Economic loss due to diseases in Indian shrimp farming with special reference to *Enterocytozoon hepatopenaei* (EHP) and white spot syndrome virus (WSSV). Aquaculture.

[3] Kim B.S., Jang G.I., Kim S.M., Kim Y.S., Jeon Y.G., Oh Y.K., Hwang J.Y., Kwon M.G. (2021). First report of *Enterocytozoon hepatopenaei* infection in Pacific Whiteleg Shrimp (*Litopenaeus vannamei*) cultured in Korea. Animals.

[4] Jang G.I., Kim S.M., Oh Y.K., Lee S.J., Hong S.Y., Lee H.E., Kwon M.G., Kim B.S. (2022). First Report of *Enterocytozoon hepatopenaei* infection in Giant Freshwater Prawn (*Macrobrachium rosenbergii* de Man) cultured in the Republic of Korea. Animals.

[5] Kim J.H., Lee C., Jeon H.J., Kim B.K., Lee N.K., Choi S.K., Han J.E. (2022). First report on *Enterocytozoon hepatopenaei* (EHP) infection in Pacific white shrimp (*Penaeus vannamei*) cultured in Korea. Aquaculture.

[6] Navaneeth Krishnan A., Jagadeesan V., Ezhil Praveena P., Bhuvaneswari T., Jithendran K.P. (2024). A comparative analysis of different challenge routes against *Enterocytozoon hepatopenaei* infection in *Penaeus vannamei*. Aquac. Int..

[7] Krishnan A.N., Kannappan S., Aneesh P.T., Praveena P.E., Jithendran K.P. (2021). Polychaete worm—A passive carrier for *Enterocytozoon hepatopenaei* in shrimp. Aquaculture.

[8] Mondal S., Deepika A., Hundare S., Poojary N., Abraham T., Sreedharan K., Rajendran K. (2023). A study on the natural host range of *Enterocytozoon hepatopenaei* in different species of shrimp and co-habiting aquatic fauna. Indian J. Agric. Res..

[9] Tanpichai P., Charoenwai O., Sataporn C., Srisuwatanasagul S., Chothirunpanit A., Suksumran N., Piamsomboon P. (2025). Experimental infection of *Enterocytozoon hepatopenaei* (EHP) by water and sediment transfer between Pacific white shrimp (*Penaeus vannamei*) and Green Mud Crab (*Scylla paramamosain*). Pak. Vet. J..

[10] Munkongwongsiri N., Thepmanee O., Lertsiri K., Vanichviriyakit R., Itsathitphaisarn O., Sritunyalucksana K. (2022). False mussels (*Mytilopsis leucophaeata*) can be mechanical carriers of the shrimp microsporidian *Enterocytozoon hepatopenaei* (EHP). J. Invertebr. Pathol..

[11] Jaroenlak P., Sanguanrut P., Williams B.A.P., Stentiford G.D., Flegel T.W., Sritunyalucksana K., Itsathitphaisarn O. (2016). A nested PCR assay to avoid false positive detection of the microsporidian *Enterocytozoon hepatopenaei* (EHP) in environmental samples in shrimp farms. PLoS ONE.

[12] Chaijarasphong T., Munkongwongsiri N., Stentiford G.D., Aldama-Cano D.J., Thansa K., Flegel T.W., Sritunyalucksana K., Itsathitphaisarn O. (2021). The shrimp microsporidian *Enterocytozoon hepatopenaei* (EHP): Biology, pathology, diagnostics and control. J. Invertebr. Pathol..

[13] Liu Y.M., Qiu L., Sheng A.Z., Wan X.Y., Cheng D.Y., Huang J. (2018). Quantitative detection method of *Enterocytozoon hepatopenaei* using TaqMan probe real-time PCR. J. Invertebr. Pathol..

[14] Sathish K.T., Praveena P.E., Sivaramakrishnan T., Rajan J.J.S., Makesh M., Jithendran K.P. (2022). Effect of *Enterocytozoon hepatopenaei* (EHP) infection on physiology, metabolism, immunity, and growth of *Penaeus vannamei*. Aquaculture.

[15] Cao Z., Chen C., Wang C., Li T., Chang L., Si L., Yan D. (2023). *Enterocytozoon hepatopenaei* (EHP) infection alters the metabolic processes and induces oxidative stress in *Penaeus vannamei*. Animals.

[16] Ma R., Zhu B., Xiong J., Chen J. (2024). The pathogenic mechanism of *Enterocytozoon hepatopenaei* in *Litopenaeus vannamei*. Microorganisms.

[17] Shen H., Dou Y., Li H., Qiao Y., Jiang G., Wan X., Cheng J., Fan X., Li H., Wang L. (2022). Changes in the intestinal microbiota of Pacific white shrimp (*Litopenaeus vannamei*) with different severities of *Enterocytozoon hepatopenaei* infection. J. Invertebr. Pathol..

